# Amino Acid Biosynthesis Pathways in Diatoms

**DOI:** 10.3390/metabo3020294

**Published:** 2013-04-18

**Authors:** Mariusz A. Bromke

**Affiliations:** Max Planck Institute of Molecular Plant Physiology, Am Mühlenberg 1, PotsdamMax Planck Institute of Molecular Plant Physiology, Am Mühlenberg 1, Potsdam 14476, Germany; E-Mail: bromke@mpimp-golm.mpg.de; Tel.: +49-331-5678247; Fax: +49-331-5678408

**Keywords:** diatoms, amino acids biosynthesis

## Abstract

Amino acids are not only building blocks for proteins but serve as precursors for the synthesis of many metabolites with multiple functions in growth and other biological processes of a living organism. The biosynthesis of amino acids is tightly connected with central carbon, nitrogen and sulfur metabolism. Recent publication of genome sequences for two diatoms *Thalassiosira pseudonana* and *Phaeodactylum tricornutum* created an opportunity for extensive studies on the structure of these metabolic pathways. Based on sequence homology found in the analyzed diatomal genes, the biosynthesis of amino acids in diatoms seems to be similar to higher plants. However, one of the most striking differences between the pathways in plants and in diatomas is that the latter possess and utilize the urea cycle. It serves as an important anaplerotic pathway for carbon fixation into amino acids and other N-containing compounds, which are essential for diatom growth and contribute to their high productivity.

## 1. Introduction

Diatoms (*Bacillariophyceae*) are a group of unicellular eukaryotic algae. Their characteristic trait is the highly ornamented siliceous cell wall called a frustule. It resembles a Petri dish since it is composed of two parts where one is slightly bigger than the other. This is also the origin of their name, as the term ‘diatom’ is derived from the Greek word *diatomos* meaning “cut in half”.

The *Bacillariophyceae* phylum is a very diverse group of organisms with conservatively estimated 100000 extant species [1]. Diatoms can be found in almost any aquatic environment. They are not only a part of the plankton in open waters, both fresh and marine, but there are many benthic species colonizing surfaces of rocks and macroalgae as well as growing in sediments. Some species can actually be both [2]. There are even species which can be found in soil [3]. Wherever there is enough humidity, light and nutrients to sustain growth, regardless whether in tropical or polar regions, there are diatoms. Generally, diatoms are regarded as photoautotrophic organisms, but there are examples of mixotrophy and even parasitism [4]. Diatoms are of profound importance to ecosystems because of their effective photosynthetic fixation of carbon and the use of inorganic nutrients. Marine diatoms are accounted for 20% of the global primary production [5,6]. Through their photosynthetic fixation of CO_2_ and the formation of organic compounds diatoms play a crucial role in maintaining the food chain in seas. Due to their relatively heavy cell wall which facilitates gravitational sinking, diatoms contribute to a flux of organic matter beneath the photic zone, which enables life in these parts of the ocean. Furthermore, diatoms contribute to biogeochemical cycling of carbon through sedimentation which precludes CO_2_ from cycling for a longer time. The sinking diatom-derived organic matter helped over millennia to form petroleum deposits [7,8].

Diatoms have a complex evolutionary history. Silica-encased diatoms evolved in Mesozoic, but remained a minor component of marine phytoplankton until Cretaceous [9]. The ancestors of diatoms have been formed through secondary endosymbiosis in which a heterotrophic cell engulfed a red algae [10]. Moreover, an endosymbiosis with a prasinophyte-like green algae (which predates the red algal capture) might explain origin of “green” genes, contribution of which is reaching 16% of the diatom proteome [11]. In consequence, diatoms carry genes of the heterotrophic host as well as of green and red algal origin. The genetic potential of diatoms was further increased by horizontal gene transfer, as genes originating from Chlamydia [12] and from other bacteria [13] can be found in genomes of the two sequenced diatoms, *Thalassiosira pseudonana* [14] and *Phaeodactylum tricornutum* [13]. One example of an unexpected attribute is that diatoms have a complete urea cycle earlier thought to occur only in Metazoa [14]. Moreover, planktonic diatoms have evolved a nutrient storage vacuole that retains high concentration of nitrate and phosphate [15] and allows diatoms to acquire inorganic nutrients coming in irregular pulses. Thus, diatoms can deprive competing taxa of these essential resources. The storage capacity of the vacuole is sufficient to allow 2–3 cell divisions without the need for external resources [9]. In consequence, diatoms can grow very well in places where nutrients are supplied in irregular pulses such as upwelling regions.

## 2. Amino Acid Synthesis Pathways in Diatoms

The recently sequenced genomes of four diatom species: The pelagic, centric *Thalassiosira pseudonana* [14]; the benthic, pennate *Phaeodactylum tricornutum* [13]; the psychrophylic *Fragilariopsis cylindrus* (Joint Genome Institute, unpublished); and the toxin-producing *Pseudo-nitzschia multiseries* (Joint Genome Institute, unpublished), provide a valuable opportunity to explore genetic background for metabolic networks in diatoms. The sequencing data of the *T. pseudonana* and *P. tricornutum* were used to reconstruct biosynthetic pathways in diatoms and the results were presented on-line in the Kyoto Encyclopedia of Genes and Genomes [16], and DiatomCyc [17]. Nevertheless, some ambiguities remain, and there are many gene sequences of unassigned function, which might represent novel biosynthetic pathways. This publication describes diatomal pathways of amino acids biosynthesis based on available sequence data.

Diatoms, like other photoautotrophs synthesize a whole range of amino acids for building proteins and other compounds such as polyamines [18], glutathione [19], heme [20], *etc.* The carbon backbones of amino acids are derived from central carbon metabolism: glycolysis (pyruvate, phosphoenolpyruvate), photosynthesis (3-phosphoglyceric acid, erythrose-4-phosphate, ribose-5-phosphate), oxidative pentose phosphate pathway (erythrose-4-phosphate, ribose-5-phosphate), photorespiration (glyoxylate, hydroxypyruvate), and TCA cycle (2-oxoglutarate, oxaloacetate) [21].

## 3. Glutamine, Glutamate, Aspartate and Alanine Biosynthesis

Before inorganic nitrogen can be incorporated into amino acids it has to be reduced to ammonia. NH_3_ can be assimilated into glutamine and glutamate through the action of glutamine synthetase and glutamate dehydrogenase, respectively. In the genomes of both *P. tricornutum* and *T. pseudonana* two isoforms of glutamine synthetase (EC 6.3.1.2) can be found. The analysis of these four sequences with HECTAR software [22] suggests that in each organism glutamine synthetase II is localized to plastids, while glutamine synthetase I is predicted to be localised to cytosol (*T. pseudonana*) and mitochondria (*P. tricornutum*). It is possible that in *P. tricornutum* glutamine synthetase I is dually localized in order to help reassmilate ammonia from the photorespiration. The mitochondrial localization of glutamine synthetase has been already reported in plants [23]. The ammonia which has been incorporated into glutamine can be used to synthesize glutamate in reaction with 2-oxoglutarate, which in *P. tricornutum* and *T. pseudonana* seems to be catalyzed by one enzymatic complex (EC 1.4.1.14 and 1.4.1.13). Moreover, ammonium can be used to form glutamate in reaction catalyzed by glutamate dehydrogenase (EC 1.4.1.4). Sequences of two isoforms of this enzyme were found in *T. pseudonana* and in *P. tricornutum*.

Aspartate synthesis uses oxaloacetate as carbon backbone. The amino group is donated by the glutamate. This reaction is catalyzed by aspartate aminotransferase (EC 2.6.1.1) and in both sequenced diatoms two isoforms of this enzyme were found. In prokaryotes, aspartate can be used to create alanine through the action of aspartate decarboxylase (EC 4.1.1.12). However, no gene sequence assigned to this enzyme could be found. It seems that the synthesis of alanine is catalyzed primarily by alanine transaminases (EC 2.6.1.2) which transfer the amino moiety from glutamate onto pyruvate. Two isoforms have been found in the genomes of both diatom species.

## 4. Glycine and Serine Biosynthesis

**Figure 1:**
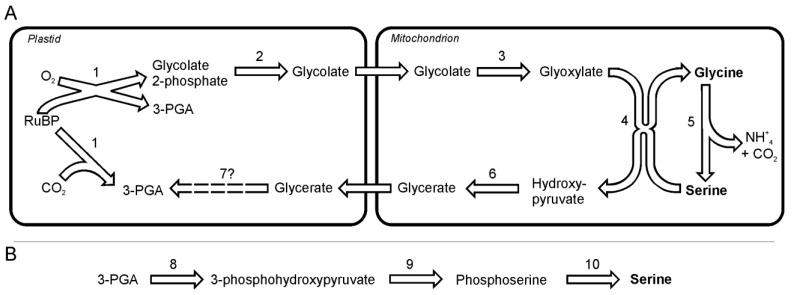
The pathway of glycine and two pathways of serine synthesis. A. Glycine and serine synthesis as part of the photorespiration. B. A non-photorespiratory pathway of serine synthesis. Reactions denoted as numbers are catalyzed by following enzymes:(**1**) ribulose-1,5-bisphosphate carboxylase oxygenase, (**2**) phosphoglycolate phosphatase, (**3**) glyoxylate synthase, (**4**) alanine:glyoxylate aminotransferase / serine:glyoxylate aminotransferase, (**5**) glycine decarboxylase + serine hydroxymethyl-transferase, (**6**) hydroxypyruvate reductase, (**7**) glycerate kinase, (**8**) 3-phoshoglycerate dehydrogenase, (**9**) phosphoserine transaminase, (**10**) phosphoserine phosphatase. Abbreviations used: 3-PGA, 3-phosphoglycerate; RuBP, ribulose-1,5-bisphosphate.

In diatoms, as in plants, serine and glycine seem to be synthesized in photorespiratory glycolate pathway [24]. The analysis of the gene sequences suggest that glycine and serine synthesis takes place in plastids and mitochondria, without the involvement of peroxisomes [24]. The photorespiration is initiated by incorporation of O_2_ into ribulose-1, 5-bisphosphate by ribulose-1,5-bisphosphate carboxylase oxygenase (EC 4.1.1.39), which yields one 3-phosphoglyceric acid and one 2-phosphoglycolate in reaction of oxygenation. The latter product cannot be used in the Calvin-Benson cycle and is instead salvaged through the photorespiratory cycle. 2-phosphoglycolate after dephosphorylation and oxidation reactions gives rise to toxic glyoxylate, which in turn is the direct precursor for glycine synthesis (Figure 1A). The first of those two reactions is catalyzed by 2-phosphoglycolate phosphatase (EC 3.1.3.18) followed by oxidation by mitochondria-targeted glyoxylate synthase (EC 1.1.3.15). Glyoxylate is transaminated to glycine probably by alanine: glyoxylate aminotransferase (EC 2.6.1.44). A part of the glyoxylate pool is transaminated to glycine via serine:glyoxylate aminotransferase (EC 2.6.1.45). Serine, which serves here as donor of the amino group, is itself synthesized from two molecules of glycine in two reactions catalyzed by glycine decarboxylase complex and serine hydroxymethyltransferase (EC 2.1.2.1). The ammonia released in this reaction can be used in the synthesis of other N-containing compounds such as glutamate or together with CO_2_ (also released in the synthesis of serine) can be used in mitochondria to form carbamoyl phosphate, which is a substrate in the urea cycle. Both enzymes can be found in genomes of the two diatoms. The in plants the subsequent conversion of serine to hydroxypyruvate and its further reduction to glycerate allows resupplying of the Calvin-Benson cycle with 3-phosphoglycerate. One should note here, that Kroth *et al.* [24] could not find any sequence of glycerate kinase in either *P. tricornutum* or *T. pseudonana*.

Both diatoms can synthesize serine in a non-photorespiratory pathway (Figure 1B) in a way similar to higher plants [25] and *Chlamydomonas reinhardtii* [26],. This pathway begins with a NADP-dependent oxidation of 3-phosphoglycerate to 3-phosphohydroxypyruvate by a 3-phoshoglycerate dehydrogenase (EC 1.1.1.95). This reaction is followed by a transamination catalyzed by phosphoserine transaminase (EC 2.6.1.52) to yield phosphoserine. Subsequently, phosphoserine is dephosphorylated to serine by the specific enzyme phosphoserine phosphatase (EC 3.1.3.3).

## 5. Cysteine Biosynthesis

Marine diatoms such as *T. pseudonana* and *P. tricornutum* live in sulfate-rich environment, where the availability of sulfur for biosynthesis of cysteine and methionine is no limiting factor. Sulfate assimilation is a relatively expensive process, as it requires eight electrons to reduce sulfate to sulfide, which can be incorporated into cysteine [27]. The reduction of SO_4_^2−^ to S^2−^ consumes 732 kJ mol^−1^. For comparison, the reduction of nitrate to NH_3_ requires also eight electrons and only 347 kJ mol^−1^ [27].

**Figure 2:**
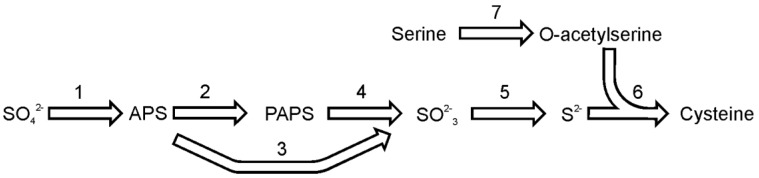
The pathway of cysteine synthesis. Reactions denoted as numbers are catalyzed by following enzymes: (**1**) ATP sulfurylase, (**2**) adenosine 5′-phosphosulfate kinase, (**3**) adenosine 5′-phosphosulfate reductase, (**4**) 3′-phospho-adenosine 5′-phosphosulfate reductase, (**5**) sulfite reductase, (**6**) O-acetyl(thiol)lyase, (**7**) serine acetyltransferase. Abbreviations used: APS, adenosine 5′-phosphosulfate; PAPS, 3′-phospho-adenosine 5′-phosphosulfate.

Sulfate reduction and the pathway of cysteine synthesis in diatoms (Figure 2), with special focus on *T. pseudonana,* has been recently reviewed by Bromke and Hesse [28]. Briefly, the sulfate reduction pathway is initiated by coupling sulfate with ATP to form adenosine-5′-phosphosulfate. This reaction is catalyzed by the enzyme ATP sulfurylase (EC 2.7.7.4). The genome of *T. pseudonana* contains two sequences that could be annotated as ATP sulfurylase. One ORF encodes for the single enzyme and the second isoform encodes a composite enzyme consisting of ATP sulfurylase fused to adenosine-5′-phosphosulfate kinase (EC 2.7.1.25) and inorganic pyrophosphatase (EC 3.6.1.1). The first enzyme activates sulfate and produces adenosine-5′-phosphosulfate (APS), while the activity of latter the composite enzyme would result in synthesis of 3'-phospho-adenosine-5′-phosphosulfate (PAPS). The composite ATP sulfurylase is also found in *P. tricornutum*. The gene sequence analysis suggests the presence of a targeting sequence in the *T. pseudonana* composite ATP sulfurylase protein, which would direct the protein into plastids; hence, sulfate in plastids could be used to synthesize 3′-phospho-adenosine-5′-phosphosulfate [28]. Adenosine-5′-phosphosulfate or 3′-phospho-adenosine-5′-phosphosulfate (the product of adenosine-5′-phosphosulfate kinase) can be reduced to sulfite by adenosine-5′-phosphosulfate reductase (EC 1.8.99.2) or 3′-phospho-adenosine-5′-phosphosulfate reductase (EC 1.8.4.8), respectively. The sequences of these enzymes of are relatively similar with the main difference between APS reductase (APR) and PAPS reductase being the presence of a [Fe4S4] cluster coordinated by two invariant cysteine pairs in almost all known APS reductases [29]. Two sequences encoding putative reductases are found in the genome of *T. pseudonana*. The analysis of protein sequence suggests that one of the APS reductases is localized in the plastid, while the other one is probably found in the cytosol [28]. Both APR enzymes from *T. pseudonana* contain a thioredoxin domain in their C- termini, in which a conserved motif CxxC can be found. The enzymes lack another pair of cysteines, which is a prerequisite for proper binding of the FeS cofactor [29], nevertheless the *T. pseudonana* protein extract reduces APS at very high rates [30,31]. Kopriva *et al.* [32] suggests that these enzymes, like the one found in mosses, belong to a novel class of APS reductases, (APR-B). The enzymes of class B lack two cysteines to bind the FeS cofactor and are able to reduce both APS and PAPS to produce sulfite. Sulfite is toxic and should be swiftly removed from a cell. This task is performed by sulfite reductase (EC 1.8.7.1) by a reaction which yields sulfide. In both *P. tricornutum* and *T. pseudonana* only single copy of sulfite reductase could be identified.

To form cysteine, sulfide is incorporated into O-acetylserine in a reaction catalyzed by the enzyme O-acetylserine (thiol)lyase (EC 2.5.1.47). The enzyme is regulated by serine acetyltransferase (EC 2.3.1.30), an enzyme that synthesizes O-acetylserine by acetylation of serine. Both enzymes, serine acetyltransferase and O-acetyserine(thiol)lyase, are necessary to build a functional cysteine synthase complex and their interaction enables an allosteric regulation of the cysteine formation [33]. In the genome of *T. pseudonana*, three genes encoding serine acetyltransferase and four O-acetylserine (thiol)lyase sequences were identified. The genome of *P. tricornutum* encodes one serine acetyltransferase and three O-acetylserine (thiol)lyase proteins.

## 6. Asparagine, Threonine, Methionine and Lysine Biosynthesis

**Figure 3:**
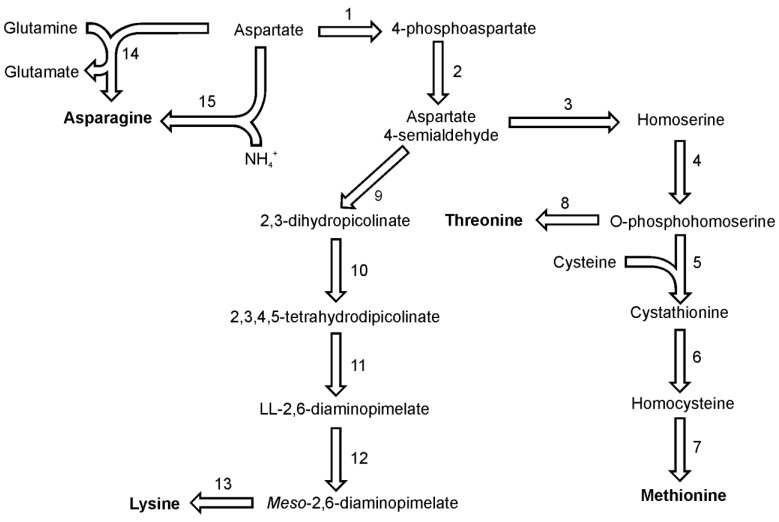
Pathways of lysine, methionine and threonine synthesis. Reactions denoted as numbers are catalyzed by following enzymes: (**1**) aspartate kinase, (**2**) aspartate semialdehyde dehydrogenase, (**3**) homoserine dehydrogenase, (**4**) homoserine kinase, (**5**) cystathionine-γ-synthase,(**6**) cystathionine-β-lyase, (**7**) methionine synthase, (**8**) threonine synthase, (**9**) dihydrodipicolinate synthase, (**10**) dihydrodipicolinate reductase, (**11**) LL-diaminopimelate aminotransferase, (**12**) diaminopimelate epimerase, (**13**) diaminopimelate epimerase, (14) asparagine synthetase, (**15**) ammonia:aspartate ligase.

Asparagine in diatoms can be synthesized from aspartate by either of two reactions, utilizing either glutamine or ammonia as the amino group donor (Figure 3). Both reactions are ATP driven and yield AMP and pyrophosphate. The first reaction is catalyzed only by the glutamine-utilizing asparagine synthetase B (EC 6.3.5.4), for which one putative sequence can be found in both genomes. The second reaction with ammonia is catalyzed by asparagine synthetase A which is also known as aspartate-ammonia ligase (EC 6.3.1.1). For this enzyme one sequence was found in the genome of each *P. tricornutum* and *T. pseudonana*. Moreover one sequence of aparaginase (EC 3.5.1.1) which catalyzes the hydrolysis of asparagine and the release of ammonia has been found in sequenced genomes both diatoms.

Threonine, together with methionine and lysine, belongs to the aspartate family of amino acids since their biosynthesis pathways start with aspartic acid (Figure 3). Threonine shares the first four steps of its synthesis with methionine. In the analyzed diatoms, the last common precursor seems to be O-phosphohomoserine. The first reaction of methionine and threonine synthesis in plants and in both diatoms is catalyzed by an aspartate kinase (EC 2.7.2.4), which forms 4-phosphoaspartate. In *T. pseudonana* two enzymes are annotated as aspartate kinase. One of them has a C-terminal domain of homoserine dehydrogenase (which probably catalyzes also one of the downstream reactions). The next step in this pathway is catalyzed by a NADPH-dependent aspartate semialdehyde dehydrogenase (EC 1.2.1.11), which forms aspartate 4-semialdehyde. The aspartate family pathway branches at this point and the carbon flux is distributed between the biosynthesis of lysine and the biosynthesis of methionine and threonine. In the following step of methionine and threonine synthesis, homoserine dehydrogenase (EC 1.1.1.3) reduces aspartate 4-semialdehyde yielding homoserine. This amino acid is not used for synthesis of proteins, but like serine its hydroxyl group can be derivatised by the transfer of an acetate or phosphate moiety. Homoserine synthesis in *T. pseudonana* can be catalyzed by one of the three homoserine dehydrogenases found in the genome as well as by the catalytic domain of one of the aspartate kinases. The last common reaction in the pathways of methionine and threonine synthesis is the phosphorylation of homoserine catalyzed by homoserine kinase (EC 2.7.1.39). Threonine then is synthesized by the catalytic action of threonine synthase (EC 4.2.3.1). Single isoforms of the latter two enzymes have been found in both diatoms.

In the methionine synthesis pathway O-phosphohomoserine can be used also by a cytosolic enzyme cystathionine γ-synthase (EC 2.5.1.48) (Figure 3). Cystathionine, the product of this reaction is used to form homocysteine by the action of cystathionine β-lyase (EC 4.4.1.8). Putative cystathionine γ-synthase and cystathionine β-lyase show sequence similarity to enzymes found in other marine algae and marine prokaryotes [34]. The genome of *T. pseudonana* posesses a gene encoding an enzyme with the same catalytic domain and also similar to proteins from parasitic eukaryotes *Leishmania sp.* and *Trypanosoma sp.* [34]. These organisms synthesize cysteine from methionine [35], which suggests that the diatomal enzyme might have the same function or act as methionine γ-lyase (EC 4.4.1.11). *P. tricornutum* has at least one sequence which could be annotated as cystathionine γ-synthase. In genomes of both diatoms, a sequence of homoserine acetyltransferase (EC 2.3.1.31) has been identified. It is not known if the acetylated homoserine is used in the synthesis of homocysteine in a reaction similar to the one found in fungi [36] or bacteria [37]. Homocysteine is the direct precursor for the *de novo*-synthesis of methionine. This last step is catalyzed by methionine synthase enzyme. There are two types of this enzyme present in diatoms. *T. pseudonana* utilizes only the efficient but cobalamin-dependent MetH type (EC 2.1.1.13), while *P. tricornutum* carries both MetH and a cofactor-independent MetE type (EC 2.1.1.14) of methionine synthase genes [38]. In this respect *P. tricornutum* is similar to *Chlamydomonas reinhardtii*, which also has both types of methionine synthase [38].

Lysine biosynthesis shares the first two reactions with the pathways of methionine and threonine synthesis (Figure 3). The last common intermediate for these pathways, aspartate 4-semiladehyde is used together with pyruvate by enzyme dihydrodipicolinate synthase (EC 4.2.1.52) to synthesize 2,3-dihydropicolinate. The following reactions include a reduction to 2,3,4,5-tetrahydrodipicolinate by dihydrodipicolinate reductase (EC 1.3.1.26) and a subsequent transamination reaction catalyzed by LL-diaminopimelate aminotransferase (EC 2.6.1.83) with glutamate serving as the amino group donor. The LL-2,6-diaminopimelate undergoes epimerisation catalyzed by diaminopimelate epimerase (EC 5.1.1.7) yielding the direct precursor of lysine, *meso*-2,6-diaminopimelate. In the final reaction of lysine biosynthesis, *meso*-2,6-diaminopimelate is used by diaminopimelate decarboxylase (EC 4.1.1.20). For all the enzymes named here a single copy of a respective gene was found in the genomes of *P. tricornutum* and *T. pseudonana*. The biochemical proof for a functional diaminopimelate pathway in pennate diatoms was delivered by Brown [39].

## 7. Valine, Leucine and Isoleucine Biosynthesis

**Figure 4:**
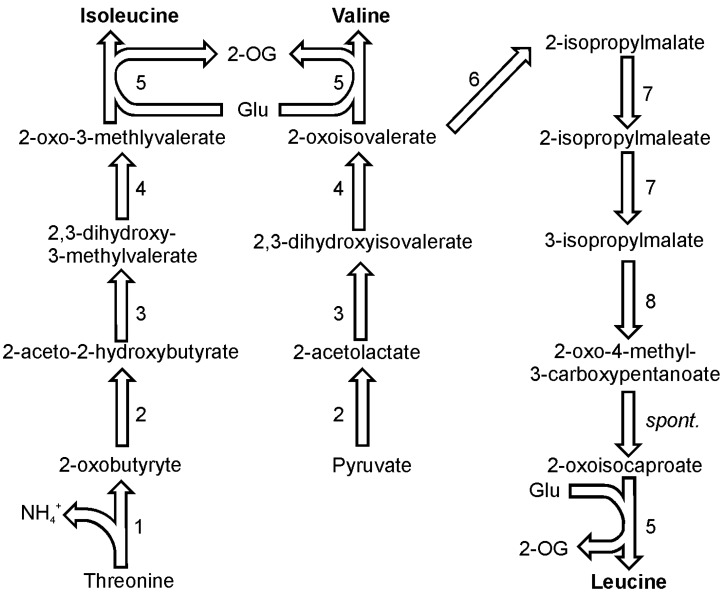
Pathways of isoleucine, valine and leucine synthesis. Reactions denoted as numbers are catalyzed by following enzymes: (**1**) threonine deaminase, (**2**) 2-aceto-2-hydroxy-butyrate synthase, (**3**) ketol-acid reductoisomerase, (**4**) dihydroxy-acid dehydratase, (**5**) branched-chain amino acid transaminase, (**6**) 2-isopropylmalate synthase, (**7**) isopropylmalate isomerase, (**8**) 3-isopropylmalate dehydrogenase. Abbreviations used: 2-OG, 2-oxoglutarate; spont., spontaneous.

In plants, isoleucine is synthesized from threonine and it seems that this pathway functions in diatoms as well [40]. The first enzyme on the pathway towards isoleucine, threonine deaminase (EC 4.3.1.19), competes with the threonine catabolism enzyme, threonine aldolase (EC 4.1.2.5). The first enzyme produces an intermediate required for isoleucine synthesis, 2-oxobutyrate, and ammonia, while the latter converts threonine to glycine and acetaldehyde (Figure 4). Threonine deaminase is present in one copy in the genomes of both discussed diatom species. Pyruvate and 2-oxobutyrate are the substrates for enzymatic activity of 2-aceto-2-hydroxy-butyrate synthase (also known as carboxy-lyase, EC 2.2.1.6) which yields one molecule of 2-aceto-2-hydroxybutanoate. This compound is in turn reduced by ketol-acid reductoisomerase (EC 1.1.1.86) giving 2,3-dihydroxy-3-methylvalerate. In *P. tricornutum* there are two ORFs thought to encode this enzyme and in the other diatom species only one copy has been found. 2,3-Dihydroxy-3-methylvaleric acid is a substrate for the activity of dihydroxy-acid dehydratase (EC 4.2.1.9) which produces the direct precursor of isoleucine 2-oxo-3-methylvalerate. Finally, a branched-chain amino acid transaminase (EC 2.6.1.42) catalyzes the transfer of an amino group from glutamate onto the oxoacid of isoleucine. In the genome of *P. tricornutum* five copies of branched-chain amino acid transaminases have been detected, while the genome of *T. pseudonana* encodes three isoforms.

The pathway of valine biosynthesis is a four-step pathway that shares all of its steps with the parallel pathway of isoleucine biosynthesis (Figure 4). The synthesis of this amino acid starts with the synthesis of 2-acetolactate from two molecules of pyruvate. 2-acetolactate is reduced by ketol-acid reductoisomerase to yield 2,3-dihydroxyisovalerate. This metabolite is used by the water-releasing dihydroxy-acid dehydratase which produces 2-oxoisovalerate. As in case of isoleucine, the final step of valine synthesis is catalyzed by a branched-chain amino acid transaminase.

2-Oxoisovalerate, which is the direct substrate for valine is also a substrate in the pathway which yields leucine (Figure 4). Four out of subsequent six reactions utilize specific enzymes, followed by one spontaneous reaction and eventually, a transamination catalyzed by a branched-chain amino acid transaminase like in case of isoleucine and valine. In both diatom genomes two genes were found to encode a 2-isopropylmalate synthase (EC 2.3.3.13). The product of the reaction catalyzed by this enzyme, 2-isopropylmalate (3-carboxy-3-hydroxyisocaproate), is used by isopropylmalate isomerase (EC 4.2.1.33), which catalyzes two reactions: dehydration and hydration. The product of the two reactions, 3-isopropylmalate (3-carboxy-2-hydroxyisocaproate), undergoes a NAD^+^-dependent oxidation catalyzed by 3-isopropylmalate dehydrogenase (EC 1.1.1.85). This reaction is followed by a spontaneous decarboxylation producing an oxoacid of leucine, 4-methyl-2-oxopentanoate. The last reaction in this pathway is catalyzed by one of the branched-chain amino acid transaminases. If not mentioned differently, all enzymes in this pathway are present as a single copy gene in the genomes of the two sequenced diatoms.

## 8. Phenylalanine, Tyrosine and Tryptophan Biosynthesis

**Figure 5:**
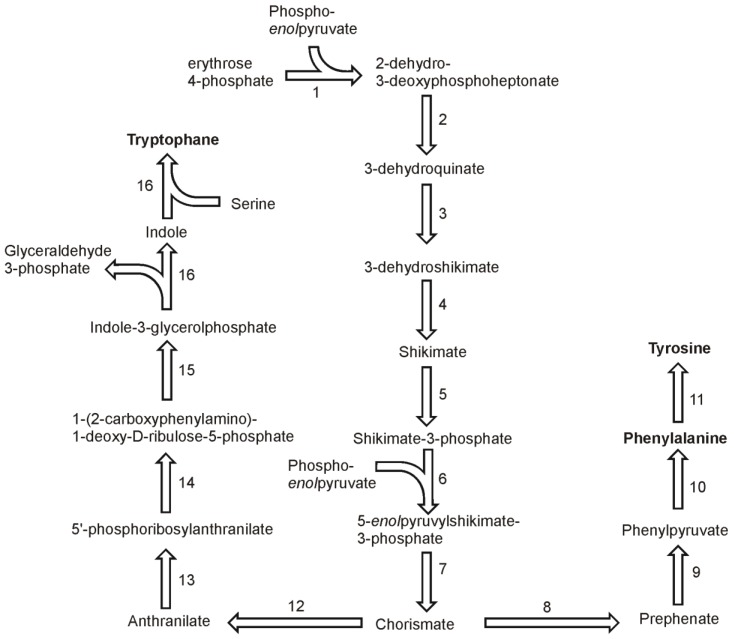
Pathways of aromatic amino acids synthesis. Reactions denoted as numbers are catalyzed by following enzymes: (**1**) 2-dehydro-3-deoxyphosphoheptonate aldolase, (**2**) dehydroquinate synthase, (**3**) 3-dehydroquinate dehydratase, (**4**) shikimate-NADP oxidoreductase, (**5**) shikimate kinase, (**6**) 5-*enol*pyruvylshikimate-3-phosphate synthase, (**7**) chorismate synthase, (**8**) chorismate mutase, (**9**) prephenate dehydrogenase, (**10**) phenylalanine:2-oxoglutarate transaminases, (**11**) phenylalanine 4-monooxygenase, (**12**) anthranilate synthase, (**13**) anthranilate phosphoribosyltransferase, (**14**) phosphoribosylanthranilate isomerase, (**15**) indole-3-glycerolphosphate synthase, (**16**) tryptophan synthase.

In higher plants and in Chlamydomonas three aromatic amino acids: phenylalanine, tyrosine and tryptophan, are derived from a common precursor, chorismate [41]. Basing on gene homology, the synthesis pathways of aromatic amino acids in diatoms appear to be similar. The synthesis of chorismate begins with condensation of erythrose 4-phosphate and phospho*enol*pyruvate in reaction catalyzed by 2-dehydro-3-deoxyphosphoheptonate aldolase (also known as 3-deoxy-7-phosphoheptulonate synthase, 2.5.1.54) (Figure 5). Two genes were found in the genome of *T. pseudonana* and one in the genome of *P. tricornutum*. The reaction product, 2-dehydro-3-deoxyphosphoheptonate is used to form 3-dehydroquinate in a reaction of cyclisation catalyzed by 3-dehydroquinate synthase (EC 4.2.3.4). Each genome has at least three sequences annotated as 3-dehydroquinate synthase genes. The third enzyme of the chorismate biosynthesis pathway, 3-dehydroquinate dehydratase (EC 4.2.1.10) convert 3-dehydoquinate into 3-dehydroshikimate, which is reduced to shikimate in the fourth step by a shikimate-NADP oxidoreductase (EC 1.1.1.25). A subsequent phosphorylation of shikimate to form shikimate-3-phosphate is catalyzed by shikimate kinase (EC 2.7.1.71). The genomes of both diatoms have a single copy of 3-dehydroquinate dehydratase and shikimate:NADP oxidoreductase. The fifth reaction in the pathway is catalyzed by an enzyme for which two copies were found in the genomes of each *T. pseudonana* and *P. tricornutum.* Sikimate 3-phosphate and phosphoenolpyruvate are used to produce 5-*enol*pyruvylshikimate-3-phosphate and orthophosphate. This reaction is catalyzed by 5-*enol*pyruvylshikimate-3-phosphate synthase (EC 2.5.1.19). Two genes for this enzyme were found in both diatomal genomes. The final step in the synthesis of chorismate, the dephosphorylation of 5-*enol*pyruvylshikimate-3-phosphate to form chorismate, is catalyzed by chorismate synthase (EC 4.2.3.5), which is encoded by only one gene in each of the diatoms.

Prephenate is the next intermediate in the synthesis pathway of tyrosine and phenylalanine. It is produced from chorismate by the action of chorismate mutase (EC 5.4.99.5) for which two genes were found in *P. tricornutum* and one in *T. pseudonana* genome. On basis of the identified genes it is likely that the pathway of phenylalanine and tyrosine goes via phenylpyruvate and not *p*-hydroxyphenylpyruvate. The other possibility is a branching to the final products after transamination of the intermediate arogenate. A gene for prephenate dehydrogenase (EC 4.2.1.51) has been found in *P. tricornutum*, which suggest the synthesis of phenolpyruvate in the diatomal cell. This compound is used by one of four phyenylalanine:2-oxoglutarate transaminases (EC 2.6.1.57) found in the *P. tricornutum* genome. Four genes of the transaminase were also found in *T. pseudonana*. In both genomes, the genes annotated as prephenate:aspartate and prehenate:glutamate transaminases were found. This fact does not exclude the functioning of an alternative synthesis pathway in which the final phenylalanine synthesis reaction is the transamination. In each diatomal genome only a single gene was found which could be annotated as a phenylalanine 4-monooxygenase (EC 1.14.16.1). Since no genes of enzymes catalyzing the synthesis of tyrosine from arogenate were found, this suggests that the oxygenation of phenylalanine is the only way of achieving tyrosine biosynthesis.

The biosynthesis of tryptophan in diatoms seems to be similar to the one found in plants, where the pathway begins with the conversion of chorismate to anthranilate by the elimination of the *enol*pyruvyl side chain by a transfer of the amino group from glutamine (Figure 5). As in other organisms, this reaction is catalyzed by the heteromeric enzyme anthranilate synthase (EC 4.1.3.27) [42]. In the next step, anthranilate phosphoribosyltransferase (EC 2.4.1.18) converts anthranilate to 5′-phosphoribosylanthranilate. In the genome of *P. tricornutum* two sequences could be annotated as the genes coding this enzyme. 5′-phosphoribosylanthranilate undergoes isomerisation by the action of the phosphoribosylanthranilate isomerase (EC 5.3.1.24). One sequence of this enzyme was found in the *P. tricornutum* genome. Following reaction of indole-3-glycerol-phosphate synthase (EC 4.1.1.48) produces indole-3-glycerolphosphate from 1-(2-carboxyphenylamino)-1-deoxy-D-ribulose-5-phosphate. There are two ORF’s for this enzyme in the genome of *P. tricornutum*. In plants, the two last steps in this pathway are catalyzed by a single enzyme, tryptophan synthase (EC 4.2.1.20), which is composed of two α and two β subunits [41]. Each of the subunits is responsible for catalyzing an individual step. Thus, the activity of tryptophan synthase can be divided into two separate steps in the pathway, each catalyzed by the corresponding subunit. First subunit cleaves glyceraldehyde-3-phosphate from indole-3-glycerolphosphate giving one indole molecule, which in turn is combined with serine giving rise to tryptophan. In *P. tricornutum* there is one gene for each of the two subunits of the tryptophan synthase complex. In contrast, *T. pseudonana* has only one ORF in which two catalytic subdomains TrpA and TrpB can be found. This is another example of a fused enzyme catalyzing subsequent reactions in this diatom. Despite the missing data on the genes of this pathway in *T. pseudonana*, one can assume that the general architecture of the pathway is as it is described for *P. tricornutum*, with the exceptions mentioned here.

## 9. Histidine Biosynthesis

**Figure 6:**
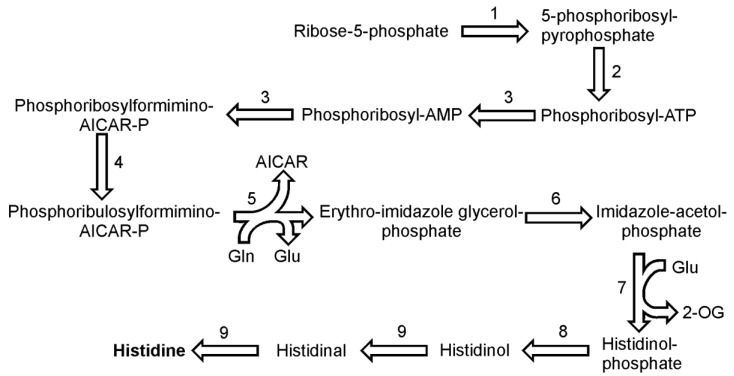
Pathway of histidine synthesis. Reactions denoted as numbers are catalyzed by the following enzymes: (**1**) ribose-phosphate diphosphokinase, (**2**) ATP phosphoribosyltransferase, (**3**) phosphoribosyl-ATP pyrophosphatase / phosphoribosyl-AMP cyclohydrolase,(**4**) phosphoribosylformiminoAICAR-P isomerase, (**5**) imidazole glycerol phosphate synthase, (**6**) imidazoleglycerol-phosphate dehydratase,(**7**) enzyme histidinol-phosphate transaminase, (**8**) histidinol phosphate phosphatase, (**9**) histidinol dehydrogenase. Abbreviations used: 2-OG, 2-oxoglutarate.

The pathway of histidine biosynthesis seems to proceed in similar way to that found in plants [43]. All genes for the enzymes involved were identified in the genome of *P. tricornutum* and all but one in case of *T. pseudonana*. The histidine synthesis begins with the ATP-dependent pyrophosphorylation of ribose-5-phosphate catalyzed by ribose-phosphate diphosphokinase (EC 2.7.6.1) to form 5-phosphoribosyl diphosphate (Figure 6). In the following reaction 5-phosphoribosyl diphosphate and ATP are bound by ATP phosphoribosyltransferase (EC 2.4.2.17) to phosphoribosyl-ATP. The product is further hydrolyzed by bi-functional enzyme phosphoribosyl-ATP pyrophosphatase (EC 3.6.1.31) / phosphoribosyl-AMP cyclohydrolase (EC 3.5.4.19) to phosphoribosyl-AMP. In the next step, the same enzyme opens the aromatic ring of adenine giving rise to N-5-phosphoribosyl-formimino-5-amino-imidazole-4-carboxamide ribonucleotide (also known as phospho-ribosylformiminoAICAR-P). One copy of this gene was found in both *P. tricornutum* and *T. pseudonana* genomes. PhosphoribosylformiminoAICAR-P further is converted by a phosphoribosylformiminoAICAR-P isomerase (EC 5.3.1.16) to phosphoribulosylformiminoAICAR-P. The following step, a conversion of phosphoribulosylformiminoAICAR-P to erythro-imidazoleglycerol phosphate and 5-aminoimidazole-4-carboxamide ribonucleotide (AICAR), requires two catalytic functions, a transamidation (with glutamine as amide donor) and a cyclization reaction, both of which are catalyzed in diatoms by the single enzyme imidazole glycerol phosphate synthase (2.4.2.-). It seems that diatoms, like plants, use the single enzyme, whereas prokaryotes use two separate enzymes to catalyze this reaction [43]. In the reaction catalyzed by imidazoleglycerol-phosphate dehydratase (EC 4.2.1.19), erythro-imidazoleglycerol phosphate produces imidazole-acetol-phosphate, which is converted to histidinol-phosphate in a subsequent reaction by an enzyme histidinol-phosphate transaminase (EC 2.6.1.9). This reaction consumes also glutamate as the amino group donor. It was long thought that there are no histidinol phosphate phosphatases in plants but recently a functional histidinol phosphate phosphatase (EC 3.1.3.15) gene has been identified in Arabidopsis [44]. The two sequences reported by DiatomCyc might be properly annotated as they both show presence of a hisB domain, which indicates a prokaryotic origin of these enzymes. The penultimate and the final reaction of histidine biosynthesis is catalyzed by histidinol dehydrogenase (EC 1.1.1.23). The enzyme catalyzes the synthesis of histidine from histidinol in two subsequent oxidation reactions with histidinal as the intermediate.

The dehydratation of erythro-imidazoleglycerol phosphate and the dephosphorylation of histidinol-phosphate can be catalyzed by two enzymes, as for each them two ORFs have been found in the genome of *P. tricornutum*. For the rest of the reactions described here, only one copy of a gene encoding respective enzyme has been found. In the genome of *T. pseudonana* each enzyme is represented by one gene and so far no histidinol phosphate phosphatases have been annotated.

## 10. Arginine and Proline Biosynthesis

**Figure 7:**
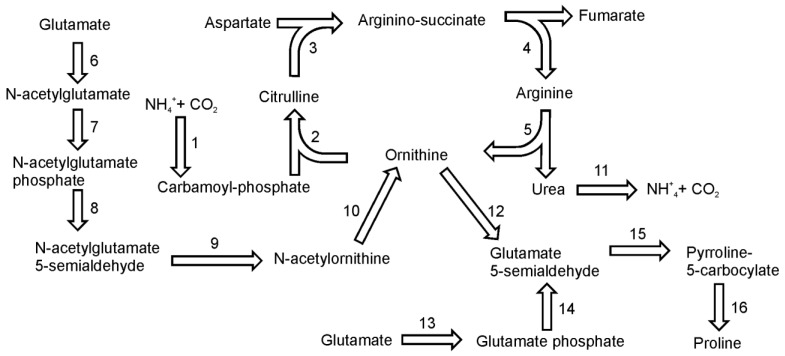
Pathways of arginine and proline synthesis. Reactions denoted as numbers are catalyzed by following enzymes: (**1**) carbamoyl-phosphate synthase, (**2**) ornithine carbamoyltransferase, (**3**) argininosuccinate synthase, (4) argininosuccinate lyase, (5) arginase, (**6**) N-acetylglutamate syntase (**7**) *N*-acetylglutamate kinase, (**8**) N-acetylglutamyl reductase, (**9**) acetylornithine transaminase, (**10**) amidoacylase, (**11**) urease, (**12**) ornithine-delta-transaminase,(**13**) + (**14**) pyrroline-5-carboxylate synthetase, (**15**) *spontaneous*, (**16**) pyrroline-5-carboxylate reductase.

Glutamate is the common precursor for the synthesis of both amino acids. In addition, ornithine, an important intermediate in the arginine biosynthetic pathway, can serve as an alternative precursor for proline biosynthesis. On the basis of gene homology there seem to be two plant-like pathways for proline biosynthesis [45]. The main pathway utilizes glutamate, which is reduced to glutamate-semialdehyde by the pyrroline-5-carboxylate synthetase enzyme (Figure 7). This conversion has two steps: glutamate phosphorylation (EC 2.7.2.11) and phosphoglutamate reduction (EC 1.2.1.41). It seems that, like plants, diatoms have only one bifunctional enzyme to catalyze this reaction. Subsequently, glutamate-5-semialdehyde is spontaneously converted to pyrroline-5-carboxylate. Pyrroline-5-carboxylate reductase (EC 1.5.1.2) further reduces the pyrroline-5-carboxylate intermediate to proline. In the genome of *P. tricornutum* three sequences were annotated as pyrroline-5-carboxylate reductase, whereas only single gene was found in *T. pseudonana*. Alternatively, proline can be synthesized from ornithine, which is first transaminated by ornithine-delta-transaminase (EC 2.6.1.13) producing glutamate-semialdehyde. Both diatoms have a single gene for this enzyme. Further reactions are the same as in the “glutamate-derived” pathway described above.

Arginine biosynthesis in diatoms is tightly connected with urea cycle in which ornithine is converted to amino acids. In diatoms and in plants ornithine synthesis is initiated by the acetylation of glutamate using acetyl-CoA [46] (Figure 5). The reaction is catalyzed by *N*-acetylglutamate synthase (EC 2.3.1.1). There are two genes for N-acetylglutamate synthase in the genome of *T. pseudonana* and one in the genome of *P. tricornutum*. The second step of the ornithine pathway is the phosphorylation of *N* -acetylglutamate by *N*-acetylglutamate kinase (EC 2.7.2.8). *N*-acetylglutamyl-phosphate then undergoes a NADPH-dependent, dephosphorylating reduction catalyzed by *N*-acetylglutamyl reductase (EC 1.2.1.38) to produce N-acetylglutamyl semialdehyde. The product of this reaction is further converted into N-acetylortnithine by acetylornithine transaminase (EC 2.6.1.11) with glutamate as the amino group donor. A subsequent reaction catalyzed by action of amidoacylase (EC 3.5.1.14) produces ornithine and acetate. Sequences of ornithine transacetylase (EC 2.3.1.35) have not been found in the genome of *T. pseudonana* or in *P. tricornutum*. This is one of the differences in metabolic pathways between diatoms and plants, where this enzyme catalyzes the synthesis of ornithine. The conversion of ornithine to arginine proceeds in three steps of the urea cycle. The genes of the urea cycle were first described in *T. pseudonana* by Armbrust *et al.* [14] and later in *P. tricornutum* by Bowler *et al.* [13]. The functionality of the cycle was confirmed biochemically by Allen *et al.* [47]. Diatoms utilize both ammonium and urea as nitrogen source for growth and therefore have no need to excrete them as waste products as in vertebrates. In diatoms, the urea cycle is an important anaplerotic carbon-fixation pathway that facilitates turnover and reallocation of intracellular carbon and nitrogen into building blocks necessary for growth – amino acids and polyamines [47]. The ammonia from the protein turnover or photorespiration together with CO_2_ is used by mitochondrial carbamoyl-phosphate synthases to form a substrate for the urea cycle – carbamoyl-phosphate. Ornithine is converted to citrulline by reaction with carbamoyl-phosphate catalyzed by ornithine carbamoyltransferase (EC 2.1.3.3). In a subsequent reaction citrulline reacts with aspartate in an ATP-dependent reaction catalyzed by argininosuccinate synthase (EC 6.3.4.5). The product of this reaction, argininosuccinate is split by argininosuccinate lyase (EC 4.3.2.1) to arginine and fumarate. In the last step of the urea cycle, arginine is cleaved by arginase (EC 3.5.3.1.) giving urea and citrulline.

## 11. Summary

The sequenced genomes of diatoms *Thalassiosira pseudonana* and *Phaeodactylum tricornutum* are valuable resources in the exploration of metabolic pathways in these ecologically important organisms. Despite their complex genetic background of diatoms, which involves at least two events of endosymbiosis and horizontal gene transfer, the general picture of amino acid biosynthesis pathways looks very similar to the one observed in higher plants or green algae. However, there are differences which make diatoms remarkable. One of them is the fully functional urea cycle, which greatly influences nitrogen metabolism and the synthesis of amino acids. Another interesting feature observed in the genomes of the discussed diatoms is the fact that enzymes catalyzing subsequent steps can be fused into one ORF like it is observed for ATP sulfurylase and tryptophan synthase. The genome sequence data are almost complete but there are still open questions concerning the details of the amino acids biosynthesis pathways. A thorough mining through the available sequence data will probably reveal more interesting examples of pathways, gene fusions, transfers and other mutations that helped diatoms to become such an abundant and successful group of organisms. Nevertheless, a pathway analysis based on homologies does not allow the understanding of the regulatory and adaptive mechanisms behind the network of reactions. These pathway predictions as well as questions of regulatory mechanisms of metabolism in diatoms will be answered with the use of molecular genetics methods.
